# Hormone-induced DNA damage response and repair mediated by cyclin D1 in breast and prostate cancer

**DOI:** 10.18632/oncotarget.19413

**Published:** 2017-07-20

**Authors:** Gabriele Di Sante, Agnese Di Rocco, Claudia Pupo, Mathew C. Casimiro, Richard G. Pestell

**Affiliations:** ^1^ Pennsylvania Cancer and Regenerative Medicine Research Center, Baruch S. Blumberg Institute, Pennsylvania Biotechnology Center, PA, USA; ^2^ Lee Kong Chian School of Medicine, Nanyang Technological University, Singapore

**Keywords:** cyclin D1, DNA damage, hormones, genomic instability, DNA repair

## Abstract

Cell cycle control proteins govern events that leads to the production of two identical daughter cells. Distinct sequential temporal phases, Gap 1 (G_1_), Gap 0 (G_0_), Synthesis (S), Gap 2 (G_2_) and Mitosis (M) are negotiated through a series of check points during which the favorability of the local cellular environment is assessed, prior to replicating DNA [1]. Cyclin D1 has been characterized as a key regulatory subunit of the holoenzyme that promotes the G_1_/S-phase transition through phosphorylating the pRB protein. Cyclin D1 overexpression is considered a driving force in several types of cancers and cdk inhibitors are being used effectively in the clinic for treatment of ERα^+^ breast cancer [1, 2]. Genomic DNA is assaulted by damaging ionizing radiation, chemical carcinogens, and reactive oxygen species (ROS) which are generated by cellular metabolism. Furthermore, specific hormones including estrogens [3, 4] and androgens [5] govern pathways that damage DNA. Defects in the DNA Damage Response (DDR) pathway can lead to genomic instability and cancer. Evidence is emerging that cyclin D1 bind proteins involved in DNA repair including BRCA1 [6], RAD51 [7], BRCA2 [8] and is involved in the DNA damage and DNA repair processes [7, 8]. Because the repair of damaged DNA appears to be an important and unexpected role for cyclin D1, and inhibitors of cyclin D1-dependent kinase activity are being used in the clinic, the latest findings on the role of cyclin D1 in mediating the DDR including the DDR induced by the hormones estrogen [9] and androgen [10, 11] is reviewed.

## INTRODUCTION

The cell cycle is defined by a series of events that lead to a production of two identical daughter cells [12, 13]. It is divided into distinct temporal phases, Gap 1 (G_1_), Gap 0 (G_0_), Synthesis (S), Gap 2 (G_2_) and Mitosis (M) [1, 13]. Cyclins include cyclin A, B, D and E. D-type cyclins are important in regulating cell cycle progression and a variety of tumorigenic processes [1]. Cell cycle progression is mainly regulated by cyclins. They specifically interact with and activate serine/threonine kinases named cyclin-dependent kinases (Cdks). Therefore, the cyclin-Cdk complexes govern a linear progression of events that lead cells from a resting state, G_0_, through to G_1_, S, G_2_ and M phases [14].

Cyclin D1 promotes the G_1_/S-phase transition by binding and activating Cdk4 and Cdk6 [15]. Strong evidence implicates cyclin D1 overexpression as a collaborative oncogene in many types of cancers. Targeted expression of cyclin D1 to the mammary gland is sufficient for the induction of mammary tumorigenesis [16, 17]. Curiously, forced expression of a cyclin D1 mutant, defective in cdk binding, is also sufficient for the induction of mammary tumors with similar latency [17, 18] suggesting additional cdk-independent functions, such as the induction of chromosomal instability [19] may contribute to the pro-oncogenic phenotype [20, 21].

Cyclin-cdk complexes phosphorylate many substrates (pRB, NRF1, GCN5) [15, 22, 23] and cyclin D1 conveys cdk-independent functions which together regulate A) gene transcription factor interactions, such as Androgen Receptor (AR) [24], Estrogen Receptor alpha (ERα) [25], Peroxisome Proliferator-Activated Receptor gamma (PPARɣ), MyoD, B-Myb, DMP1 [26-29] and retinoblastoma (Rb) B) cellular metabolism through Nuclear Respiratory Factor 1 (NRF1) [22]; c) fat cell differentiation, through C/EBPβ and PPARɣ [30, 31] and d) cellular migration [32, 33]. Cyclin D1 regulates nuclear hormone receptors including the AR [24] and ERα [34, 35] both in tissue culture , and *in vivo* [25, 36]. Cyclin D1 enhances transcription of estrogen responsive genes in a kinase independent activity. Cyclin D1 selectively inhibits ligand-dependent AR function in several cell types, including breast cancer, bladder cancer, and androgen-independent prostate adenocarcinoma cell lines. Cyclin D1 forms a specific complex with the AR, requiring the C terminus of cyclin D1 [15].

Over the last three decades several different studies have assessed the role of cyclin D1 in DNA damage repair. Genomic DNA is continually subjected to insults by damaging ionizing radiation, chemical carcinogens, and reactive oxygen species (ROS) generated by cellular metabolism. Defects in the DDR can lead to genomic instability and cancer [10].

Given the key role for DNA Damage Response (DDR) and subsequent repair process in maintaining genomic integrity understanding the role of cyclin D1 in DNA damage and repair is important and is reviewed herein. Subsequent studies have focused on the role of cyclin D1 in the DNA damage repair associated with hormonal signaling. In this regard both estrogen and androgen signaling have been linked to DNA damage, and defective DNA repair has been linked to malignancies of both breast and prostate cancer. Herein, we summarize the latest findings on the role of cyclin D1 in mediating hormone-induced DDR.

## THE DNA DAMAGE RESPONSE IN CANCER

Thousands of DNA lesions occur per hour in each cell of the human body. In general, the DNA damage can be considered as either endogenous or exogenous in origin. The endogenous damages can be generated by reactive oxygen species (ROS) produced during normal cell metabolism or accumulation of replication errors during the DNA replication process. On the other hand, external agents, such as ultraviolet radiation, x-rays, gamma (γ) rays, mutagenic chemicals, plant toxins and viruses are responsible for exogenous damage. DNA damage can have deleterious effects, as it interferes with DNA replication and transcription, causing mutations and chromosomal aberrations. Genome integrity is preserved by the DNA damage repair machinery, which counteracts the adverse consequence of DNA lesions and prevents their transmission to daughter cells [37].

The DNA damage response (DDR) is a collective term for all intra- and inter-cellular signaling events and enzyme activities that result from the induction and detection of DNA damage. These include events that lead to cell-cycle arrest, regulation of DNA replication, and the repair or bypass of DNA damage [38]. DNA damage can induce a DNA break in a single strand (SSBs) or a double strand (DSBs). SSBs form upon oxidative attack of deoxyribose by cell metabolism products or through exposure to agents such as H_2_O_2_, ionizing radiation, and radiomimetic drugs. They also arise as intermediates during excision repair of damaged bases, and upon inhibition of topoisomerase I. In most eukaryotes, SSBs are initially detected by PARP-1, whose binding to DNA breaks triggers poly-ADP-ribosylation of nuclear proteins [39]. DSBs are generated in response to ionizing radiation or radiomimetic drugs by free radical attack of deoxyribose, and also arise in cells treated with topoisomerase II inhibitors. DSBs can be generated by other factors, including mechanical stress of chromosomes, DNA polymerase encountering a SSB and other DNA lesions [40]. This form of DNA lesion is most hazardous because it can lead to genomic rearrangement.

There are two major forms of repair when dealing with DNA double-strand breaks (DSBs): Homologous recombination repair (HRR) and non-homologous end-joining (NHEJ) pathways [41]. HRR is a relatively accurate and efficient repair pathway but depends upon the presence of an undamaged sister chromatid [42], while NHEJ pathways (Classical-NHEJ and alternative-NHEJ, C-NHEJ and alt-NHEJ respectively) are not dependent on the presence of a sister allele and, while still effective, are less accurate, potentially introducing DNA rearrangements [43, 44]. BRCA1 participates is homologous recombination, where the repair proteins utilize a template of the identical homologous intact sequence from a sister chromatid, from a homologous chromosome, or from the same chromosome, depending on cell cycle phase. BRCA1 also participates in termed mismatch repair. The BRCA1 protein interacts with RAD51 during repair of DNA double-strand breaks and co-localizes with γ-H2AX (histone H2AX phosphorylated on Serine-139) in DNA double-strand break repair foci. The BRCA2 protein, which has a function similar to that of BRCA1, also interacts with the RAD51 protein. RAD51 is a 339-amino acid protein that plays a major role in homologous recombination of DNA during double strand break repair. In this process, an ATP-dependent DNA strand exchange takes place in which a template strand invades base-paired strands of homologous DNA molecules.

It has been reported that the major transducers involved in DDR are ataxia-telangiectasia mutated (ATM) and ATM- and Rad3-Related (ATR). ATM is activated by DSBs and triggers a cascade of events on the chromatin flanking DSBs. Upon DNA damage, ATM is activated by phosphorylation of Serine 1981, which causes dissociation of ATM homodimer [45]. Activated ATM is able to phosphorylate substrates such as p53 and H2AX. The histone variant H2AX is phosphorylated at Serine 139 (γH2AX). Mdc1 directly binds to γH2AX and is required for complete γH2AX foci formation [46-48]. Foci formation facilitates the recruitment of additional DNA repair components. In contrast to ATM, ATR responds to a broad spectrum of DNA damages, including DSBs and a variety of DNA lesions that interfere with replication. ATR is essential for the survival of proliferating cells and its deletion leads to early embryonic lethality in mouse and lethality of human cells [49-52].

E2F1 is induced in response to various DNA-damaging agents, including ionizing radiation, UV radiation, and a number of chemotherapeutic drugs [53]. Moreover, as shown by ultraviolet (UV) laser micro-irradiation and immunofluorescence staining, E2F1 accumulates at sites of DNA damage [54]. The mechanism by which E2F1 translocates to DSB lesions has been relatively well characterized showing that DNA topoisomerase II-beta protein (TopBP1) interacts with the E2F1 N-terminal domain and thereby enables E2F1 recruitment to DNA lesions [55].

It has also been reported that the action of DNA repair factors is tightly coordinated with cell cycle progression. For example, cells in G_1_ do not have an available sister chromatid and will therefore be dependent upon NHEJ pathways for the repair of DSBs. In addition, there are important differences in the primary role of checkpoints during stages of the cell cycle and in the participating DDR factors. For example, the G_1_/S checkpoint allows repair of DNA damage prior to the start of DNA replication, in order to remove obstacles to DNA synthesis, and key DDR factors regulating this checkpoint include ATM, CHK2, and p53. The intra-S phase checkpoint proteins ATR, CHK1, DNAPK, and WEE1 can delay replication origin firing to provide time to deal with any unrepaired DNA damage, thus preventing under-replicated DNA regions being conveyed beyond S-phase. The activities of the G_2_/M checkpoint proteins including CHK1, MYT1, and WEE1 lead to an increase in phosphorylated Cdk1, thereby maintaining an inactive state and delaying mitotic entry. The G_2_/M checkpoint represents the last major opportunity for preventing the passage of DNA lesions into mitosis, where unrepaired DSBs and under-replicated DNA may result in mitotic catastrophe and cell death [56]. This is of major importance since the DNA lesions promote genomic instability [57]. The cells possess a specific response mechanisms to avoid both accumulation of DNA damage and maintenance of genomic stability [58].

In contrast to normal cells, a universal hallmark of cancer cells is genomic instability due to defects in the mechanisms responsible for repair of DNA damage [57]. The pro-survival activities of DDR are realigned and misappropriated during cancer progression by activation of oncogenes such as Ras, Akt and Myc, which enhance replicative stress [57]. Replicative stress is defined as the harmful effect of DNA that is only partially replicated because of slow progression of the replication forks, which can be caused by oncogene-induced hyper-replication that activates multiple origins of replication per S phase, by nucleotide pool imbalance or by DNA damage [59]. This leads to increased DNA damage, which eventually increases genomic instability to a level that is incompatible with cell survival. However, induction of replicative stress by hyperactive growth factor signaling and oncogene signaling in established cancer can lead to compensatory upregulation of DNA repair pathways providing a new opportunity in cancer therapy [57, 59].

Historically, treatment of cancer by radiotherapy and DNA-damaging chemotherapy are based on the strategy of inducing DNA damage, promoting chromosomal catastrophe of transformed cells and ultimately the induction of cancer cell death. However, these approaches lead to significant collateral damage to normal tissue and unwanted side effects. Targeted therapy based on inhibiting DDR in cancers offers the potential for a greater therapeutic window by tailoring treatment to patients with tumors lacking specific DDR mechanisms [38].

## DNA DAMAGE RESPONSE AND CYCLIN D1

In early studies cyclin D1 was shown to restrain the DNA repair process [60]. During repair DNA synthesis, subsequent to UV-induced DNA damage, G_1_ cells readily lost their cyclin D1 while proliferating cell nuclear antigen (PCNA) tightly associated with nuclear structures. Microinjection of cyclin D1 antisense accelerated DNA repair, whereas overexpression of cyclin D1 prevented DNA repair and the relocation of PCNA after DNA damage The abundance of cyclin D1 has been shown to play a role in cell-type dependent radiation-induced sensitivity. *Cyclin D1*^*−/−*^ MEFs have enhanced apoptosis evoked by γ-irradiation [61] and cyclin D1 expression also inhibited UV induced apoptosis [62]. In contrast breast cancer cell lines overexpressing cyclin D1 showed enhanced apoptosis in response to γ irradiation [63, 64] suggesting cell type specific differences governing cyclin D1 mediated apoptosis. However, DNA damage induced rapid degradation of cyclin D1 [61]. These studies were consistent with a model in which cyclin D1 was considered to be a collaborative oncogene, and that restraining DNA repair would be consistent with such a function.

**Figure 1:**
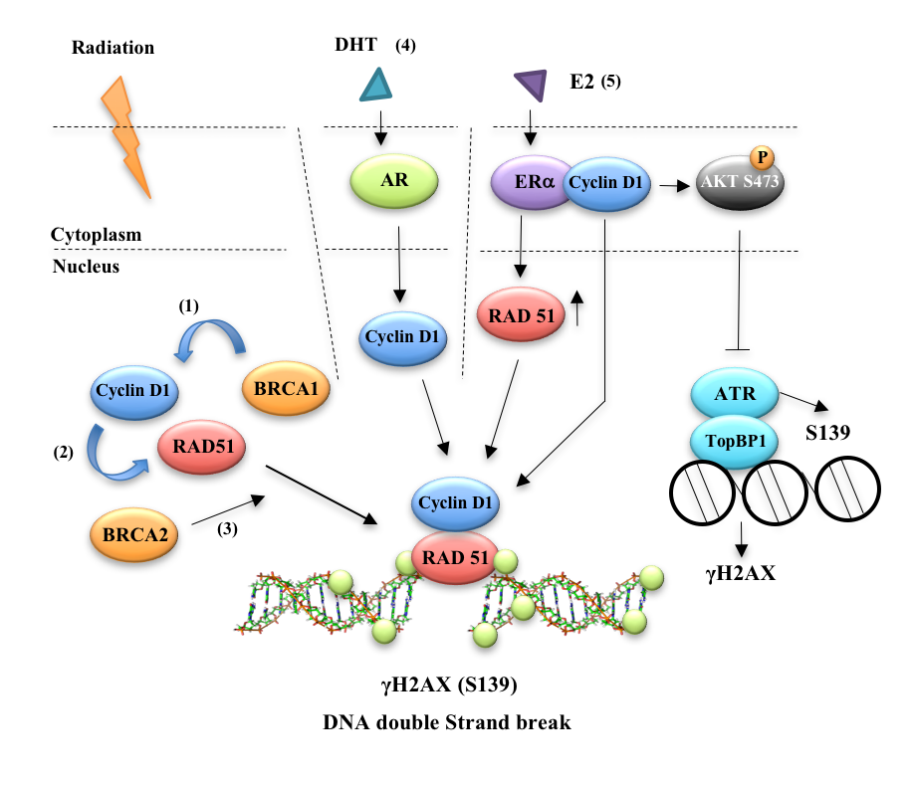
Cyclin D1 participates in estrogen and androgen mediated DNA damage repair responses Cyclin D1 binds (1) BRCA1 [6], (2) RAD51 ([7, 8]) and (3) BRCA2 [8]. Cyclin D1 was required for the (4) androgen-mediated DNA damage response both *in vitro* and *in vivo* suggesting hormone-mediated recruitment of cyclin D1 to sites of DDR may facilitate the resistance of prostate cancer cells to radiotherapy [10]. Silencing cyclin D1 leads to an impairment of DNA DSBs repair and a physical interaction was identified between cyclin D1 and activated ATM, DNA-PKcs and RAD51. Taken together these data suggest a key role for cyclin D1 in regulating the androgen-dependent DDR response [11]. (5) Using estrogen dendrimers that are excluded from the nucleus, Li et al showed that E2-mediated cyclin D1 dependent DDR occurred via a novel extra-nuclear function. Their findings revealed that ERα-cyclin D1 binding at the cytoplasmic membrane augmented AKT phosphorylation (Ser473) and γH2AX foci formation. In the nucleus, cyclin D1 enhanced homology-directed DNA repair. Cyclin D1 was recruited to γH2AX foci by E2 and induced Rad51 expression [9].

Over the subsequent decade however it has become apparent that distinct pools of cyclin D1 exist within the cell, with distinct binding partners, that govern distinct functions [20, 65, 66]. Thus, the total abundance of cellular protein may not reflect an important role for cyclin D1 in a local site, such as chromatin with a transcription factor, or at focal contacts [67]. In 2005, cyclin D1 was shown to bind to BRCA1, both in subcellular foci and in the context of transcriptional regulatory complexes in chromatin (Figure 1) [6]. As noted above, BRCA1 functions as a scaffold protein organizing DNA damage sensors, thereby coordinating the repair of damaged DNA [68-70] The *BRCA1* gene product, a nuclear polypeptide of 220 kDa, co-localizes in nuclear foci with RAD51, BRCA2, and RAD50. protein complexes that regulate repair of DNA double-strand breaks, including hRAD50-hMre11-NBS1, MSH2, MSH6, and proliferating cell nuclear antigen, functionally interact with BRCA1 [71].

In 2010, cyclin D1 was shown to enhance the DNA damage response, and the mechanisms involved were characterized [7]. RAD51 is a key protein governing homologous DNA repair. RAD51 is involved in the search for homology and strand pairing stages of the process. This protein can interact with the ssDNA-binding proteins RPA, BRCA2, PALB2 and RAD52 a recombinase that drives the homologous recombination process. The studies by Li *et al* showed that the abundance of cyclin D1 determined the DNA damage response, as characterized by induction of γH2AX phosphorylation, the assembly of RAD51 DNA repair foci, and specific recruitment of DNA repair factors to chromatin. Secondly, Li showed, that cyclin D1 bound to RAD51. Thirdly it was shown that cyclin D1 recruited RAD51 and DNA repair factors in response to DNA damage, thereby enhancing γH2AX phosphorylation. Fourthly, these studies showed that cyclin D1, like DNA repair factors, elicited the DDR when stably associated with chromatin [7]. Cyclin D1 recruited DNA repair factors in a cyclin D1 isoform specific, occurring with the cyclin D1a but not the cyclin D1b isoform. As these two isoforms vary primarily in the carboxyl terminus these studies suggested a role for the cyclin D1a isoform C terminus in these events. Fifth, the enhancement of DNA damage induced γH2AX phosphorylation by cyclin D1 involved specific kinases (DNA-PK, JNK and Casein Kinase 2). These studies demonstrated the importance of cyclin D1a in the DDR using distinct activators of the DDR, including γ irradiation, double stranded-DNA damage inducing agents (doxorubicin, 5-FU) and the targeting of single DNA repair factors to chromatin.

In 2011, a proteomic screen for cyclin D1 protein binding partners by Jirawatnotai et al, identified RAD51 and many other binding proteins. These studies extended our understanding of cyclin D1 in the DDR by showing the recruitment of cyclin D1 to sites of DNA damage was BRCA2-dependent [8]. Furthermore, these studies showed that cyclin D1 regulated not only the DNA damage response and the creation of DNA damage repair foci, but showed that cyclin D1 enhanced DNA repair [8]. Given that cyclin D1 is thought to be a collaborative oncogene, and the DNA repair defects often are associated with predisposition to cancer, it was considered “surprising, that this protein also mediates the repair of damaged DNA, a mechanism that commonly prevents cancer” [72].

These functional studies [7, 8] were however consistent with several gene expression studies in which cyclin D1 expression was shown to induce DDR gene expression. Genome wide microarray analysis demonstrated cyclin D1 induced expression of genes involved in DNA replication and DNA damage checkpoints suggesting a role for cyclin D1 in the DDR. Cyclin D1a induced the mRNA expression of Minichromosome maintenance (MCM)3, MCM4, replication factor C (activator 1) 4 (Rfc4), cell division cycle 6 homology (Cdc6), cell division cell associated 7(Cdc7) and H2Afx (H2A Histone family member x) in MEFs [73]. Mammary gland targeted cyclin D1 inducible antisense transgenic demonstrated endogenous cyclin D1 maintained expression of MCM2, Rfc2, Cdc20, Rad51 and histone 1 in the mammary epithelium [74]. In subsequent studies forced expression of cyclin D1 induced expression of genes regulating the DDR, including MCM3 and Cdc7 [75]. Expression were increased in mammary tumors derived from mammary epithelial cell targeted cyclin D1a transgenic mice. Furthermore, forced expression of a degradation defective cyclin D1a mutant induced the DDR in murine lymphoid tissue [76]. Thus, prior indirect evidence implicated cyclin D1 in enhancing gene expression governing the DDR in a number of different cell types

## HORMONALLY-MEDIATED DNA DAMAGE RESPONSE

Steroid hormones are systemically circulating small molecules that elicit autocrine, paracrine, and endocrine functions in physiology and pathology, most often through the binding to, and regulation of cognate nuclear receptors (NR) [77]. Steroid hormones influence distinct and important cancer-associated phenotypes, including, but not limited to cellular proliferation, apoptosis, migration, and invasion. Steroid-induced biological outcomes occur in a context-dependent manner in multiple malignancies, including breast (BrCa) and prostate (PCa) cancers. As such, therapy for selected hormone-dependent cancers focuses on diminishing availability of ligand or direct antagonism of NRs.

While the biological implications remain uncertain, steroid hormones (e.g., estrogens, androgens, glucocorticoids) have also been associated with the induction of genotoxic stress via multiple mechanisms, such as formation of DNA adducts, or generation of reactive oxygen species (ROS). These effects can be exacerbated by both genetic aberrations, such as mutation of important DNA repair genes, as well as chemical perturbations, or through the use of NR antagonists. Conflicting data exists as to whether hormones are carcinogenic or cancer-protective, and the discrepancies are context-, model-, and hormone-specific. However, steroid hormones have been shown to promote transformation, as well as generate complex genomic rearrangements through induction of DSBs that are associated with transformation. Conversely, hormone signaling has also been shown to stabilize DNA and be chemopreventive. Additionally, some NR antagonists (e.g., tamoxifen) have also been demonstrated to induce DNA damage, adding further complexity [77].

Estrogen is necessary for the physiological growth and development of breast tissue. Increased levels of estrogens are one of the major risk factors for breast cancer. It has been shown that estrogens can contribute to breast cancer by inducing DNA damage [4]. High levels of estrogens in serum are associated with a 2–to 2.5- fold greater risk of breast cancer [4]. Early breast cancer lesions exhibit chromosomal instability and aneuploidy, and the induction of DNA damage by estrogen leads to double stranded DNA breaks and genomic instability. Estrogen serves as a ligand for ERα, inducing nuclear receptor activity and also participates in an acute cytoplasmic membrane-associated activity (Reviewed in [78]). The ERα regulates nuclear gene expression via binding to both canonical DNA and non-canonical DNA sequences in the promoter of target genes. Extra-nuclear pools of ERα have been identified in the plasma membranes [79]. Estrogen can induce DNA damage via the production of oxidative metabolites that cause DNA adducts, or other oxidative DNA damage, evidenced by *in vitro* and animal model studies [4]. Secondly, hyper-activated estrogen signaling provokes excessive proliferation when pathways become dysregulated, and this theory has strong support from *in vitro* modeling and gene signatures in breast cancer [80]. Excessive proliferation promotes DNA damage accumulation due to insufficient timely repair, leading to replication fork stalling and possibly even double stranded DNA breaks [81]. Furthermore, E_2_ is known to delay the assembly and prolongs the resolution of γH2AX and RAD51 foci through inhibition of ATR kinase signaling [3].

Knowing that cyclin D1 interacted with the estrogen receptor, Li *et al.* examined the role for cyclin D1 in E2-mediated DNA damage signaling (Figure 1). These studies showed that cyclin D1 governs an essential role in the E2-dependent DDR in human breast cancer cells [9]. The ability to distinguish nuclear from extra-nuclear ERα signaling has been enabled through the generation of 17β-estradiol dendrimer conjugates (EDC), which are localized to the extra-nuclear compartment [82]. Using estrogen dendrimers that are excluded from the nucleus, Li *et al* showed that E2-mediated cyclin D1 dependent DDR occurred via a novel extra-nuclear function. Their findings revealed that ERα-cyclin D1 binding at the cytoplasmic membrane augmented AKT phosphorylation (Ser473) and γH2AX foci formation. In the nucleus, cyclin D1 enhanced homology-directed DNA repair. Cyclin D1 was recruited to γH2AX foci by E2 and induced RAD51 expression [9]. Given the pro-survival and proliferative function of Akt, the augmentation of Akt1 phosphorylation by cyclin D1 may contribute to aberrant growth signaling and is consistent with *in vivo* studies illustrating the importance of cyclin D1 in E2-mediated growth signaling *in vivo* [25].

Recent studies have shown androgens restrain DNA damage and enhance DNA repair [5, 83]. Sawyers et al showed that prostate cancer cells treated with IR plus androgen demonstrate enhanced DNA repair and decreased DNA damage and furthermore that antiandrogen treatment causes increased DNA damage and decreased clonogenic survival of prostate cancer cells [5]. Antiandrogen treatment resulted in decreased classical non-homologous end joining. These studies began with an unbiased query of gene expression in a clinically validated xenograft model of castration-resistant prostate cancer. The authors defined an “AR-associated DNA repair gene” signature of 144 DNA repair genes that were significantly associated with canonical AR output [5]. In a related study, DNA damage induced repair, was delayed in cultured cells deprived of androgens. Furthermore, the AR recruitment to the *XRCC2* gene after γ irradiation was said to be delayed. A 20% induction of DNA-PKcs was observed after 24 hours and phosphorylation of DNA-PKcs (Ser2056) was reduced by enzalutamide, leading to a proposed model in which liganded AR governs DNA repair via DNA-PKcs phosphorylation [83]. Curiously although it was proposed γ irradiation induced AR signaling, KLK3/PSA was not induced, suggesting further complexities remain to be understood [83]. Nonetheless together these studies were consistent with a model in which androgens enhance the repair of damaged DNA.

Like androgens, cyclin D1 promotes prostate cellular proliferation [36]. Cyclin D1 binds to the androgen receptor [24] and augments prostate stem cell expansion [36]. Furthermore, the cyclin D1-mediated gene expression signature predicts poor outcome in human prostate cancer [36]. The role of cyclin D1 in androgen-dependent signaling was therefore assessed [10]. Casimiro *et al.* found that endogenous cyclin D1 diminished further the dihydrotestosterone (DHT)-dependent reduction of γH2AX foci. They also showed that cyclin D1 was required for the androgen-mediated DNA damage response both *in vitro* and *in vivo*. These findings suggest that the hormone-mediated recruitment of cyclin D1 to sites of DDR may facilitate the resistance of prostate cancer cells to radiotherapy (Figure 1) [10]. In support of these studies, Marampon F. et al. found that silencing cyclin D1 leads to an impairment of DNA DSBs repair. Furthermore, they showed a physical interaction between cyclin D1 and activated ATM, DNA-PKcs and RAD51. Taken together these data suggest a key role for cyclin D1 in regulating the androgen-dependent DDR response [11].

The last decade has demonstrated an important role for cyclin D1-dependent kinase inhibitors in the treatment of patients with ERα^+^ breast cancers who are concurrently taking other medications, such as Fulvestrant [1, 2]. At the same time, new evidence has accrued that cyclin D1 plays an important role in the DNA damage response to a variety of therapeutic agents. Furthermore, the hormone-mediated DNA damage effects of estradiol and androgen directly involves cyclin D1. Together these studies suggest careful timing of multiple therapies may be important in optimizing therapeutic outcomes. The finding that distinct domains of cyclin D1 participate in the pRB phosphorylation function, *vs* the transcription factor interaction domains raises the possibility that targeting of multiple distinct domains of cyclin D1 may potentially also improve therapeutic specificity.
